# An App-Based Workplace Mindfulness Intervention, and Its Effects Over Time

**DOI:** 10.3389/fpsyg.2021.615137

**Published:** 2021-04-22

**Authors:** Yizhen Lu, Julien Remond, Michael Bunting, Remus Ilies, Neha Tripathi, Jayanth Narayanan

**Affiliations:** ^1^Department of Management and Organization, National University of Singapore, Singapore, Singapore; ^2^Awakened Mind, Singapore, Singapore

**Keywords:** mindfulness, well-being, growth curve analysis, organizational behavior, employee engagement, emotional exhaustion

## Abstract

We investigated the week-to-week effects of a mindfulness intervention on emotional exhaustion, work engagement, and job satisfaction in a field study involving 218 participants who participated and reported their weekly outcomes during the 8-week program. To examine how mindfulness impacted work outcomes, we used intraindividual modeling of the 8-week data. Mindfulness increased over time, and time also had indirect effects on emotional exhaustion, work engagement, and job satisfaction, through mindfulness. Supplementary growth curve analyses on the improvement of mindfulness over time showed a slight decrease in the positive effect of time on mindfulness.

## Introduction

Over the past decades, mindfulness research in organizations has been gaining traction as both scientific researchers and practitioners have reported various benefits of mindfulness (Brown et al., 2015; Creswell, 2017). The rising interest in these mindfulness-based interventions shed light on how these interventions have the potential to improve well-being of employees in the workplace (Flaxman and Bond, 2010; Aikens et al., 2014; Hülsheger et al., 2015). Other workplace intervention studies on mindfulness-based stress reduction programs found positive outcomes in employees such as job satisfaction and engagement at work (Hülsheger et al., 2013; Leroy et al., 2013). Although the evidence that mindfulness can be beneficial to workplace outcomes is increasing, given the proliferation of mindfulness programs at the workplace, it is unclear what benefits these programs bring and how these effects occur over time (Allen et al., 2015; Jamieson and Tuckey, 2017). We focus on this question and evaluate the effects of an 8-week mindfulness intervention with a specific emphasis on improving work outcomes.

### Mindfulness as an Intervention

Mindfulness intervention studies at the workplace are rare. In Virgili (2015)’s review on mindfulness-based interventions, he included studies that were not exclusive to workplace interventions. In addition, the reviewed studies predominantly used perceived stress scale as an outcome measure of the intervention (e.g., Wolever et al., 2012). Most of the studies were measured in a pre- and postintervention cross-sectional format (e.g., Newsome et al., 2012; Leroy et al., 2013; Hyland et al., 2015; Kadziolka et al., 2016). In addition, Glomb et al. (2011) noted that the intervention programs were done in clinical settings or with college students and not employees. Among workplace intervention studies, it was found to reduce perceived stress (Michel et al., 2014; Shonin et al., 2014), increase resilience at work (Aikens et al., 2014). In recent years, more research on mindfulness intervention adopted randomized controlled trials with control conditions (cf. Malouf et al., 2017; Zheng et al., 2020). In Bartlett et al.’ (2019) review, they found that the recent workplace intervention studies showed and supported past research on how mindfulness intervention increased various well-being related outcomes like sleep (Crain et al., 2016), psychological distress (Huang et al., 2015), and burnout (Moody et al., 2013; Ancona and Mendelson, 2014). Even among these studies, the effects of time have not been studied.

The present research intends to examine the development of state mindfulness over time and how the development of state mindfulness could benefit work-related outcomes like reducing emotional exhaustion and promoting work engagement and job satisfaction. Furthermore, the study design of the current mindfulness intervention program was conducted through a smartphone mindfulness application, which is novel feature of this research since few studies have focused on the remote delivery of mindfulness intervention programs (Boettcher et al., 2014; Lim et al., 2015; Krusche et al., 2020).

### Outcomes of Mindfulness

Baer et al. (2006) argued that mindfulness is multidimensional in nature, containing a set of skills that complement each other: acting with awareness of one’s actions, observing emotional and sensory experiences, being able to describe emotions and experiences in words, being non-reactive to emotions especially negative ones, and being able to suspend judgment about events. Bishop et al. (2004) refer to mindfulness as the ability to have non-judgment attentional focus and awareness of momentarily experiences. The meaning of mindfulness has been distilled into two main ideas: Firstly, mindfulness is a state of awareness in which individuals are attentive to present events in a receptive, non-reactive, and non-judgmental way. Secondly, mindfulness helps one attend to the present moment and develops an open and accepting attitude toward the event (Brown et al., 2007). According to mindfulness-to-meaning theory, these mechanisms underlying mindfulness allows for the process of decentering which helps the mindful individual to have broadened perspective toward the present (Garland et al., 2015).

In contrast to an individual’s daily experience and tendency to mind-wander, mindfulness was found to improve one’s well-being (Brown and Ryan, 2003; Creswell and Lindsay, 2014). The improvement on well-being could be the reappraisal process that is induced by mindfulness (Garland et al., 2015). Research on mindfulness has also shown benefits to individuals in various domains. In chronic pain research, mindfulness was found to be effective in pain management for chronic pain patients (Kabat-Zinn et al., 1985; Davis et al., 2015), and results in improvement in immune response (Davidson et al., 2003). Mindfulness training has also been shown to improve attentional awareness (Dane, 2011). For instance, mindfulness is associated with noticing the loss of active awareness of the present, i.e., mind wandering and automatic piloting (Mrazek et al., 2012). Similarly, mindfulness practices build a sense of openness and acceptance toward present thoughts and feelings, and these characteristics of mindfulness were shown to be helpful for individuals experiencing symptoms of depression and anxiety (Evans et al., 2008; Flaxman et al., 2013; Strauss et al., 2014; Eisendrath et al., 2016). Other well-being benefits include improvement of interpersonal functioning like stress buffering and perspective taking, which are important contributors to higher quality of interpersonal relationships (Carson et al., 2004; Karremans et al., 2017).

Mind-wandering on the other hand is associated with unhappiness (Killingsworth and Gilbert, 2010). As much as individuals are naturally prone to mind-wandering (Kabat-Zinn, 2003), it is also the case that every individual has a certain capacity to pay attention to the surroundings, to be mindful from time to time. Kiken et al. (2015) found that mindfulness training that increases state mindfulness eventually enhances one’s trait mindfulness. This capacity, in mindfulness intervention research, was found to be malleable as individuals could be trained to pay mindful attention to present moment experiences (Farb et al., 2014; Creswell, 2017). Therefore, we hypothesize the following for our smartphone mindfulness intervention field study:

*Hypothesis* 1: At the within-individual level, weekly mindfulness will increase over time.

Shapiro et al. (2006) clarified the meta mechanisms behind mindfulness practices: that mindfulness is based on reperceiving and reappraisal process that guides the shift in perspective such that the individual is able to see negative events from an objective and less emotional perspective (Kabat-Zinn, 2003; Reb et al., 2015). This process encourages cognitive based coping and reappraisal, hence reducing the experienced stress from the negative event despite being exposed to it (Cooper and Dewe, 2008). In addition, mindfulness helps individuals to develop an accepting and non-judgemental perspective toward their experiences, making it an integral part of improving emotional regulation and psychological well-being (Hülsheger et al., 2013; Lindsay and Creswell, 2015; Slutsky et al., 2019). According to affective events theory (Weiss and Cropanzano, 1996), mindfulness may help individuals to appraise workplace events positively and hence reduce negative events as stressors. Hence, when mindfulness is higher, emotional exhaustion will be reduced. When the negative events are appraised less negatively, mindfulness helps individuals to understand the situation in a non-judgemental, accepting way, which may facilitate adaptive responses instead of overwhelming them. Experiencing the negative events with acceptance effectively reduces its negative effects on the individual (Siegel and Siegel, 2014). Therefore, individuals who are low on mindfulness may find work stressors more negative than mindful individuals, and hence will experience higher emotional exhaustion. Hence, we posit that:

*Hypothesis* 2a: At the within-individual level, weekly mindfulness will be negatively related to weekly emotional exhaustion.

Similarly, mindfulness may be positively influencing individual work engagement due to its ability to build an accepting mindset and be less susceptible to distractions at work such that it improves work engagement (Leroy et al., 2013; Malinowski and Lim, 2015). Mindfulness helps to reperceive negative events such that it has less of an impact on the individual. Furthermore, according to self-determination theory, awareness from mindfulness training may help individuals focus on behaviors that helps them to meet their needs, which may increase their work engagement (Deci and Ryan, 2004). Focus achieved through the help of mindfulness by deviating from “autopilot,” which increases awareness around one’s values to regulate behaviors to achieve individual goals (Brown and Ryan, 2003; Shapiro et al., 2006). Therefore, individuals with higher levels of mindfulness will experience higher work engagement due to their ability to focus on directed behaviors. Conversely, individuals with lower levels of mindfulness will experience lower work engagement. Therefore, we hypothesize the following:

*Hypothesis* 2b: At the within-individual level, weekly mindfulness will be positively related to weekly work engagement.

**Figure 1:**
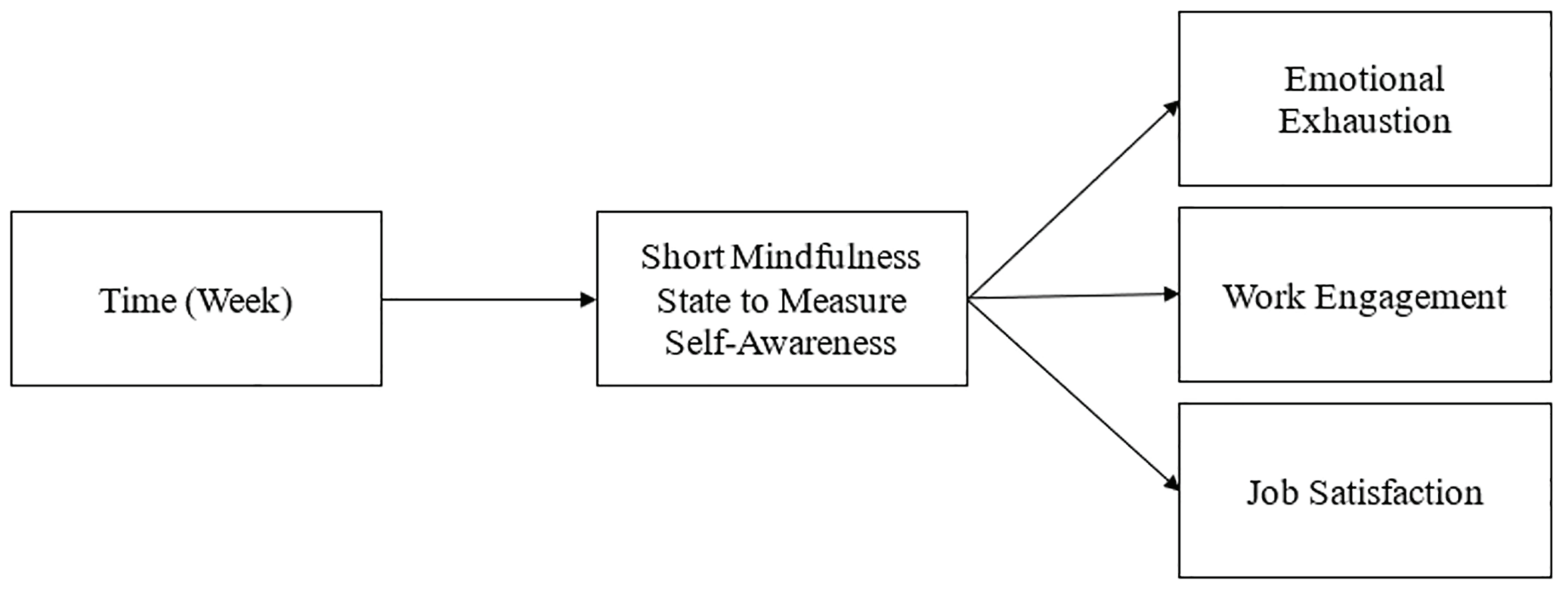
Hypothesized conceptual model.

Mindfulness influences the emotional and cognitive processing of individuals by allowing them to focus on the present moment and surrounding environment without tying emotional reactions to it (Brown and Ryan, 2003). This allows for higher receptiveness and openness to challenges faced at work. This basis of mindfulness shields the individual from accumulation of stressors. Fredrickson et al. (2008) found positive emotions to be an outcome of mindfulness interventions which in turn increased life satisfaction and lower levels of depression. Hence, we posit that higher mindfulness is related to higher levels of job satisfaction due to their ability to manage stressors better. Individuals who are lower on mindfulness may find it more difficult to handle stressors and hence may experience lower job satisfaction. For a summary of the research hypotheses, refer to Figure 1 for the conceptual model.

*Hypothesis* 2c: At the within-individual level, weekly mindfulness will be positively related to weekly job satisfaction.
*Hypothesis* 3: Within individuals, time (weeks) will have indirect effects on (a) emotional exhaustion, (b) work engagement, and (c) job satisfaction through mindfulness.

## Materials and Methods

### Sample and Procedure

For this study, 221 participants were recruited as users who signed up to use a mindfulness meditation application, Awakened Mind®. The participants range from countries of different geographical locations (i.e., Australia, India, Indonesia, New Zealand, and the United States). The application was introduced to their respective companies and they were told that a free 8-week intervention program was available for new users, and they can choose to sign up for the program with other users across the globe. All participation in the study was voluntary. Participants were working executives who had signed up for an 8-week mindfulness program on the Awakened Mind® platform. The participants did not receive monetary compensation or other incentives from participating in the program. The participant sample consisted of 50.5% females. About 58.9% of these participants have at least a bachelor’s degree, and 52% have been with their current company for at least 3 years.

**Figure 2:**
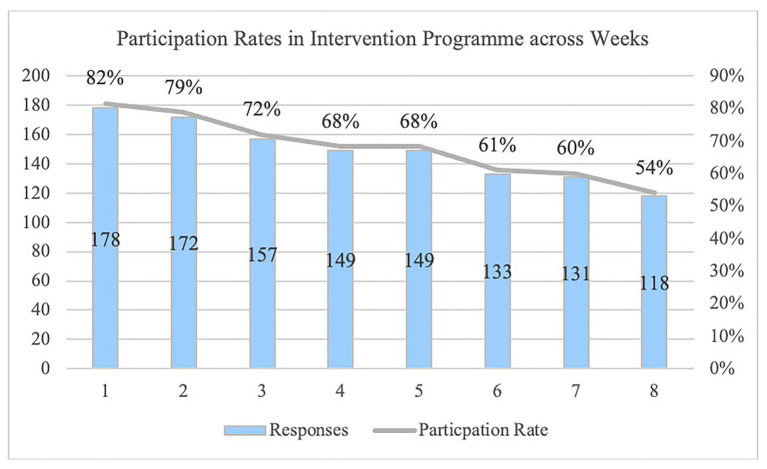
Participation rate during the intervention program.

Before the 8-week long mindfulness meditation intervention program, they were asked to respond to a baseline demographic survey (age, gender, tenure, and occupation). During the 8-week program period, the participants had access to weekly mindfulness practice and self-practice activities in the application. After each session, the participants were asked to self-report the weekly variables. The mindfulness intervention program includes attending to body sensations, stretching, and relaxation exercises in a guided audio clip. At the end of each weekly mindfulness meditation session, participants completed a survey reporting their work engagement, job satisfaction, emotional exhaustion, and mindfulness over the past week. From the weekly surveys, the overall response rate was 67% out of 1,768 possible responses. The responses were only collected if they complete the module for the week, as they could only access the survey at the end of the module. The response rate was reasonably high because of the introduction of the program to clusters of employees who are from the same company, and hence partaking in the program was part of their employee wellness in their respective companies. Refer to Figure 2 for the participation information. In sum, the participants spent a total of 6 h over 8 weeks to complete the intervention program. They were sent weekly reminders at the start of each week to make progress on their weekly modules.

#### Intervention

Participants were introduced to an 8-week mindfulness intervention program consisting of four modules. To manage the intervention program and data collection remotely, facilitators from their respective companies were assigned to 22 clusters of participants, with each cluster ranging from a team size of 4 to 16 individuals. They were guided and reminded to complete their modules and weekly surveys.

Awakened Mind® was the software of choice as it conducts the intervention using scientific methods and educational-oriented content for participants to learn more about mindfulness while practising it. A quick outline of each module is as follows: Module 1 includes an introductory program to mindfulness meditation, breathing, and relaxation techniques. Module 2 introduces fundamentals of mindfulness and mindlessness (i.e., mind wandering) and concept of acceptance. Module 3 involves attentional awareness and body scanning exercises. Module 4 includes mindfulness and how the techniques of mindfulness could be adapted to overcome potential workplace challenges. The current study was conducted using Awakened Mind® as the modules include attentional qualities of mindfulness and the practice of acceptance without evaluation or interpretation of events occurring in the external environment (Baer, 2003; Shapiro et al., 2006). In addition, the participants who signed up to use the application were highly likely to commit to the intervention program, as they were going through the program with their colleagues and supervisors. The program is standardized across individuals as it is downloadable content that will only be marked as complete when the participants tune in to the entire training session. This ultimately addresses existing concerns about mindfulness interventions delivered in organizations that were found to lack standardization and scientific content (Crane et al., 2017).

### Measures

The data collection included a demographic survey prior to the start of the intervention program. At the end of each week, the participants received email reminders to complete a weekly survey.

#### Mindfulness

The short mindfulness scale was adapted from the Mindful Attention Awareness scale (Brown and Ryan, 2003). They were given a truncated 5-item scale to assess their weekly mindfulness. Sample items include “I was doing something automatically, without being aware of what I was doing,” “I was finding it difficult to stay focused on what was happening,” and “I was rushing through something without being really attentive to it.” The items were scored such that higher values indicate high levels of mindfulness. They were asked to rate it on a 6-point Likert scale ranging from 1 = “almost always” to 6 = “almost never.”

#### Emotional Exhaustion

A 9-item scale were used for emotional exhaustion component from the Maslach burnout inventory (Maslach and Jackson, 1986). Sample items included “I feel fatigued when I get up in the morning and have to face another day on the job,” “Working with people directly puts too much stress on me,” and “Working with people all day is really a strain for me.” Emotional exhuastion was rated on a Likert scale ranging from 1 = “strongly disagree” to 5 = “strongly agree.”

#### Work Engagement

Work engagement was measured with a 5-item truncated scale ranging from 1 = “strongly disagree” to 5 = “strongly agree” (Schaufeli et al., 2002). Sample items included “When I get up in the morning, I feel like going to work,” “At my work, I feel that I am bursting with energy,” and “I feel happy when I am working intensely.”

#### Job Satisfaction

Job satisfaction was measured with a 3-item scale ranging from 1 = “strongly disagree” to 5 = “strongly agree” (Judge et al., 2000). Items included “All in all I am satisfied with my job,” “In general, I do not like my job,” and “In general, I like working here.”

### Analyses

Since the hierarchical data structure of weekly reports were in nested form, we tested the hypotheses with multilevel structural equation modeling (MSEM) using MPlus Version 7.4 (Muthén and Muthén, 2015) with time and the eight measures of each construct at the first level of analysis and individuals at the second level. This analysis strategy allows intraindividual and interindividual variability in the intraindividual change within a model of antecedents and consequents (Preacher et al., 2008). In additional analyses, to examine the growth trajectory of individuals’ the mindfulness during the intervention program, we utilized a multilevel growth model analysis (e.g., Ng et al., 2010; Leroy et al., 2013).

Prior to testing hypotheses, the relative variance in study variables was examined for its intraclass correlation values (Bliese and Ployhart, 2002). It was found that all study variables had intraclass correlation coefficient (ICC) ranging between 0.56 and 0.67, suggesting that 56–67% of variance was explained by between-person variation, whereas 33–44% of variance was explained by within-person variation, making the dataset suitable for testing within-individual effects over time.

## Results

Means, SDs, reliability, and intercorrelation between study variables are displayed in Table 1. In our confirmatory factor analyses, the data fit our measurement (five-factor) model [*χ*^2^(20) = 751.92, *p* < 0.01; CFI = 0.99; RMSEA = 0.02, SRMR_within_ = 0.035, SRMR_between_ = 0.002]. As shown in Table 2, the five-factor model showed best fit to the data compared to alternative models. Table 3 shows the indirect effects between time (weeks) and emotional exhaustion, work engagement, and job satisfaction through mindfulness. Weekly mindfulness has a significant positive relationship with time (*γ* = 0.14, *p* < 0.01), showing an improvement in mindfulness over time. Therefore, Hypothesis 1 was supported.

**Table 1:** Mean, SD, reliability, and intercorrelation of study variables.

		Mean	SD	ICC	% Var	1	2	3	4	5
1	Short mindfulness state to measure self-awareness	4.71	0.73	0.575	0.425	(0.958)				
2	Work engagement	3.45	0.69	0.563	0.437	0.262^**^	(0.956)			
3	Job satisfaction	3.65	0.68	0.672	0.328	0.171^**^	0.531^**^	(0.945)		
4	Emotional exhaustion	2.36	1.04	0.624	0.376	−0.345^**^	−0.344^**^	−0.346^**^	(0.946)	
5	Time (week)	-	-	-	-	0.249^**^	0.120^**^	0.072^**^	−0.017	-

*N* = 218. *k* = 1,187. Cronbach’s alpha was calculated for each week and averaged. ICC, intraclass correlation coefficient. Correlations are within-individual correlations.

** *p* < 0.01.

**Table 2:** Discriminant validity of study variables: multilevel confirmatory factor analyses.

Model description	*χ*^2^	*df*	CFI	RMSEA	SRMR_within_
Five-factor model	751.92	99	0.99	0.02	0.04
Four-factor model: combine job satisfaction with work engagement	843.27	102	0.88	0.07	0.08
Four-factor model: combine job satisfaction with emotional exhaustion	1388.04	102	0.79	0.09	0.09
Four-factor model: combine work engagement with emotional exhaustion	1381.98	102	0.79	0.09	0.10
Three-factor model: combine job satisfaction, work engagement, and emotional exhaustion	1422.79	104	0.78	0.09	0.10
Two-factor model: combine job satisfaction, work engagement, emotional exhaustion, and short mindfulness state to measure	2616.43	105	0.59	0.12	0.14
One-factor model: combine all variables	2598.96	104	0.59	0.12	0.14

CFI, comparative fit index; RMSEA, root mean square error of approximation; SRMR, standardized root mean square residual. *N* = 218. Number of observations = 1,187–1,744.

**Table 3:** Indirect effects.

	−1 SD		+1 SD		Δ	
	*γ* (S.E)	95% CI	*γ* (S.E)	95% CI	*γ* (S.E)	95% CI
Time ® Short mindfulness state to measure self-awareness → Emotional exhaustion	−0.07^**^ (0.02)	(−0.11, −0.03)	−0.05^**^ (0.01)	(−0.08, −0.03)	0.02^**^ (0.01)	(0.01, 0.03)
Time → Short mindfulness state to measure self-awareness → Job satisfaction	0.02^**^ (0.01)	(0.01, 0.03)	0.02^**^ (0.00)	(0.00, 0.02)	−0.01^**^ (0.00)	(−0.01, −0.00)
Time → Short mindfulness state to measure self-awareness → Work engagement	0.04^**^ (0.01)	(0.02, 0.06)	0.03^**^ (0.01)	(0.01, 0.04)	−0.01^**^ (0.00)	(−0.02, −0.00)

*N* = 187. Number of observations = 1,187.

** *p* < 0.01.

Table 4 also shows the results of a multilevel analysis investigating the direct relationships between mindfulness and emotional exhaustion, work engagement, and job satisfaction. Weekly mindfulness was found to be significantly related to emotional exhaustion (*γ* = −0.46, *p* < 0.01), work engagement (*γ* = 0.25, *p* < 0.01), and job satisfaction (*γ* = 0.14, *p* < 0.01). Hence, Hypotheses 2a–c were all supported.

**Table 4:** Multilevel regression analyses: time predicting weekly mindfulness, emotional exhaustion, work engagement, and job satisfaction.

	Short mindfulness state to measure self-awareness		Emotional exhaustion		Work engagement		Job satisfaction	
	*γ*	S.E.	*γ*	S.E.	*γ*	S.E.	*γ*	S.E.
**Within person level**								
Constant	4.33^**^	0.08	2.68^**^	0.10	3.28^**^	0.06	3.57^**^	0.06
**Predictor variables**								
Time (week)	0.14^**^	0.03	−0.10^**^	0.04	0.05^*^	0.03	0.01	0.02
Short mindfulness state to measure self-awareness	-	-	−0.46^**^	0.05	0.25^**^	0.04	0.14^**^	0.03
Residual variances	0.51**	-	1.06^**^	-	0.47^**^	-	0.45**	-
**Between person level**								
Constant	4.33^**^	0.08	5.75^**^	0.45	1.80^**^	0.40	2.07^**^	0.41
**Predictor variables**								
Short mindfulness state to measure self-awareness	-	-	−0.74^**^	0.10	0.35^**^	0.08	0.35^**^	0.09
Residual variances	0.31^**^	-	0.51^**^	-	0.23^**^	-	0.27^**^	-

*N* = 187. Number of observations = 1,187. S.E., standard error. Two-tailed test. Gammas are unstandardized.

* *p* < 0.05

** *p* < 0.01.

**Figure 3:**
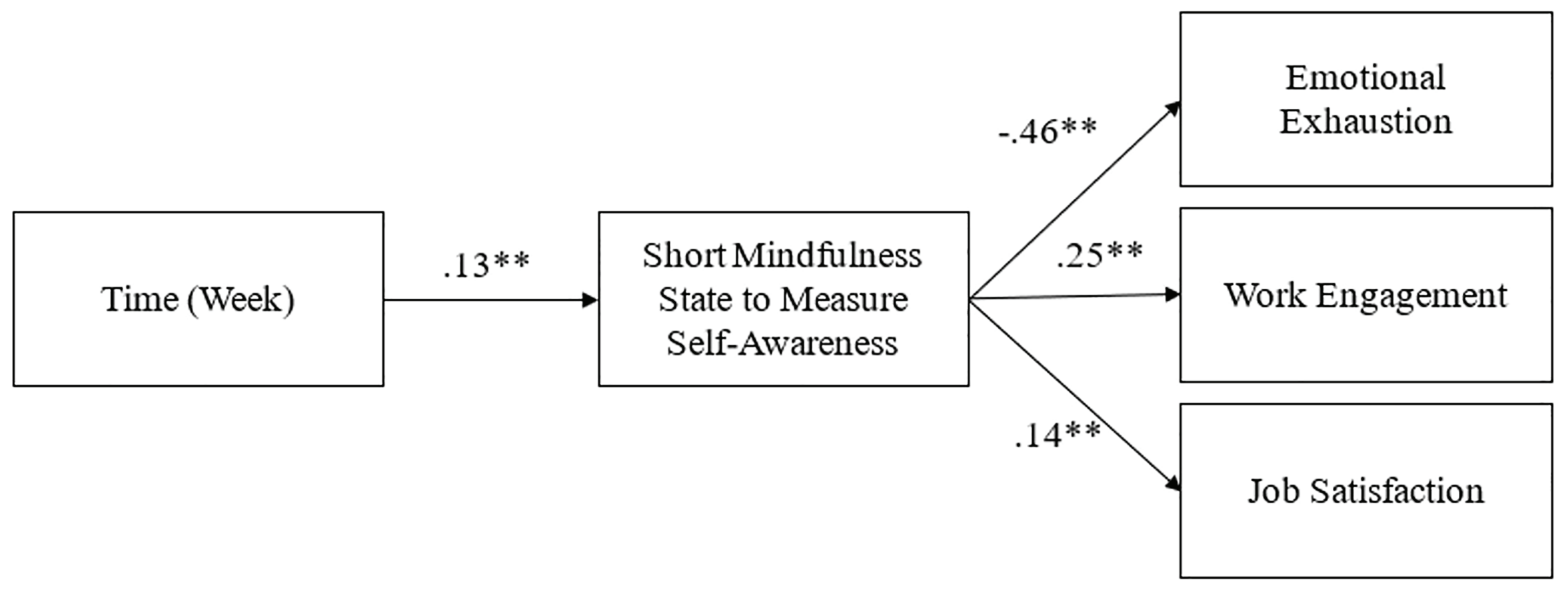
Results of hypothesized model with path analyses. ^**^p < 0.01.

Table 3 shows significant indirect effects of time on the reduction of emotional exhaustion, and on the enhancement of work engagement and job satisfaction through mindfulness. There were significant indirect effects of time on (a) emotional exhaustion (*γ* = −0.06, s.e. = 0.02, 90% CI [−0.09, −0.04]), (b) work engagement (*γ* = 0.03, s.e. = 0.01, 90% CI [0.02, 0.05]), and (c) job satisfaction (*γ* = 0.02, s.e. = 0.01, 90% CI [0.01, 0.03]) through state mindfulness. Therefore, Hypotheses 3a–c were supported. Refer to Figure 3 for path analysis results.

### Supplementary Analysis

To explore whether the effects of the mindfulness intervention program were diminishing or accelerating during the 8-week period, we ran a curvilinear test on the growth trajectory of participants. These results are shown in Table 5. We found that the changes in mindfulness had a slight downward negative curve (*γ* = −0.01, *p* < 0.05), i.e., there was a lower rate of increase in mindfulness toward the end of the program. Yet, as we predicted in the first hypothesis, time had a strong overall (linear) effect on mindfulness (also showed in Table 5).

**Table 5:** Curvilinear growth curve modeling results.

	MAAS	Work engagement	Job satisfaction	Emotional exhaustion
Constant	4.33^**^ (0.08)	3.30^**^ (0.06)	3.54^**^ (0.07)	2.63^**^ (0.10)
MAAS	-	0.10 (0.16)	−0.05 (0.15)	−0.28 (0.23)
Time	0.14^**^ (0.03)	0.05 (0.03)	0.03 (0.03)	−0.10^*^ (0.04)
Time*Time	−0.01^**^ (0.00)	0.00 (0.00)	0.00 (0.00)	0.01 (0.01)
Variance	0.051^**^ (0.05)	0.47^**^ (0.04)	0.45^**^ (0.04)	1.01^**^ (0.07)

*N* = 187. Number of observations = 1187.

* *p* < 0.5

** *p* < 0.01.

## Discussion

Current study results confirmed that the 8-week mindfulness intervention program increased weekly mindfulness. Weekly mindfulness then mediated the relationship between time and emotional exhaustion, work engagement, and job satisfaction. Results showed that mindfulness mediates the relationship between the progression of the intervention program and both well-being and workplace outcomes. The current study extends the literature on mindfulness research using a standardized mindfulness intervention program which improves mindfulness over time and hence result in well-being outcomes. As the sample was derived from organizations that sent their work teams to participate in mindfulness meditation, the format of having work teams undergoing the mindfulness practices together seemed to contribute to the positive outcome of the intervention. Furthermore, managers may consider implementing mindfulness-based intervention programs involving the work groups as designed in this piece of research to encourage participation and immersion in the intervention program. This may further encourage lower dropouts and higher level of participation in the program, compared to traditional delivery of mindfulness practices that purely consists of instructor-to-participant delivery format.

### Limitations and Future Research Directions

As the sample was recruited from new users of the mindfulness application, they were asked to test the mindfulness software and engage in mindfulness-based practices. Hence, we did not have a control group. As emphasized by Creswell’s (2017) review on mindfulness interventions, randomized experiments are key to showing promising effects of mindfulness interventions and increasing the robustness of the programs. In recent years, experiments with randomized controlled trials are becoming increasingly common with treatment or other wellness activities as usual as control conditions as comparison group (see de Bruin et al., 2016; Malouf et al., 2017; Josefsson et al., 2019). Hence, we conducted growth curve modeling as an analytical strategy to examine within-person developments during the program. In future research, randomized experiments could be used to compare within-individual trajectories between those who receive a mindfulness intervention and those who do not. Furthermore, work diaries could be collected based on events at work, to understand how positive and negative events influence the individual and how mindfulness could manage these emotions across time.

Although curvilinearity analyses indicated that rate of increase in mindfulness during the intervention program diminished slightly, participants still experienced a relatively strong linear increase in mindfulness over time. Despite the curvilinear results, we argue that the positive emotions from mindfulness may help to build personal resources that in turn improve the outcomes of the mindfulness intervention program (Fredrickson et al., 2008). This is further supported by Mindfulness-to-Meaning Theory suggested by Garland et al. (2015), that mindfulness promotes eudaimonic well-being that could support this upward positive spiral. Future research could help to address how this upward positive spiral may manifest during the intervention.

## Data Availability Statement

The raw data supporting the conclusions of this article will be made available by the authors, without undue reservation.

## Ethics Statement

The studies involving human participants were reviewed and approved by NUS Departmental Ethics Review Committee. The patients/participants provided their written informed consent to participate in this study.

## Author Contributions

YL, RI, and JN designed the research. JR oversaw the data collection. JR and MB designed the mindfulness intervention. YL and NT performed the data analysis with inputs from JN and RI. YL and JN wrote the manuscript with inputs from RI. All authors contributed to the article and approved the submitted version.

### Conflict of Interest

JR and MB run Awakened Mind as a commercial organization. However, they were not part of the analysis or the conclusions of the study.

The remaining authors declare that the research was conducted in the absence of any commercial or financial relationships that could be construed as a potential conflict of interest.
